# Vascular Structure Identification in Intraoperative 3D Contrast-Enhanced Ultrasound Data

**DOI:** 10.3390/s16040497

**Published:** 2016-04-08

**Authors:** Elisee Ilunga-Mbuyamba, Juan Gabriel Avina-Cervantes, Dirk Lindner, Ivan Cruz-Aceves, Felix Arlt, Claire Chalopin

**Affiliations:** 1 Telematics (CA), Engineering Division (DICIS), University of Guanajuato, Campus Irapuato-Salamanca, Carr. Salamanca-Valle km 3.5 + 1.8, Com. Palo Blanco, Salamanca, Gto. 36885, Mexico; e.ilungambuyamba@ugto.mx; 2 Department of Neurosurgery, University Hospital Leipzig, Leipzig 04103, Germany; Dirk.Lindner@medizin.uni-leipzig.de (D.L.); Felix.arlt@medizin.uni-leipzig.de (F.A.); 3 CONACYT Research-Fellow, Center for Research in Mathematics (CIMAT), A.C., Jalisco S/N, Col. Valenciana, Guanajuato, Gto. 36000, Mexico; ivan.cruz@cimat.mx; 4 Innovation Center Computer Assisted Surgery (ICCAS), University of Leipzig, Leipzig 04103, Germany; Claire.Chalopin@medizin.uni-leipzig.de

**Keywords:** cT1MR, 3D-iCEUS, neurosurgery, vascular structure identification, J0101

## Abstract

In this paper, a method of vascular structure identification in intraoperative 3D Contrast-Enhanced Ultrasound (CEUS) data is presented. Ultrasound imaging is commonly used in brain tumor surgery to investigate in real time the current status of cerebral structures. The use of an ultrasound contrast agent enables to highlight tumor tissue, but also surrounding blood vessels. However, these structures can be used as landmarks to estimate and correct the brain shift. This work proposes an alternative method for extracting small vascular segments close to the tumor as landmark. The patient image dataset involved in brain tumor operations includes preoperative contrast T1MR (cT1MR) data and 3D intraoperative contrast enhanced ultrasound data acquired before (3D-iCEUS${}_{start}$) and after (3D-iCEUS${}_{end}$) tumor resection. Based on rigid registration techniques, a preselected vascular segment in cT1MR is searched in 3D-iCEUS${}_{start}$ and 3D-iCEUS${}_{end}$ data. The method was validated by using three similarity measures (Normalized Gradient Field, Normalized Mutual Information and Normalized Cross Correlation). Tests were performed on data obtained from ten patients overcoming a brain tumor operation and it succeeded in nine cases. Despite the small size of the vascular structures, the artifacts in the ultrasound images and the brain tissue deformations, blood vessels were successfully identified.

## 1. Introduction

**Figure 1:**
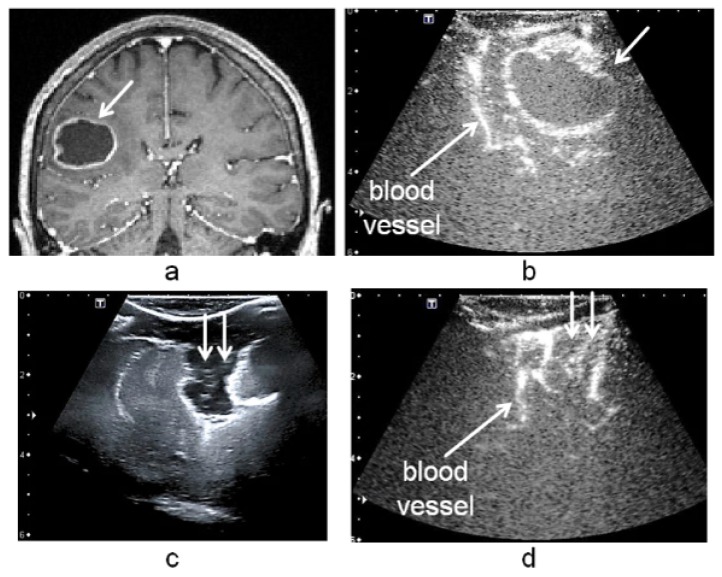
Patient image data acquired during tumor resection. The brain tumor (white arrow) is represented in the preoperative cT1MR data (**a**) and in the 3D-iCEUS${}_{start}$ (**b**) acquired at the beginning of the operation. After tumor removing, the resection cavity (indicated by two white arrows in (**c**) and (**d**) is well visible in the B-mode ultrasound image (**c**). In the 3D-iCEUS${}_{end}$ acquired at the end of the operation (**d**); the borders of the cavity can be easily interpreted as a blood vessel.

Intraoperative ultrasound imaging is nowadays commonly used in neurosurgery during brain tumor operations [1]. At the beginning of the intervention, the ultrasound images show the surgeon the intraoperative state of the tumor (Figure 1b) [2]. The tumor size or position can be possibly slightly different at the operation time point from the preoperative state depicted in the preoperative MR data (Figure 1a). During the operation ultrasound imaging is a valuable tool to detect the residuals of tumor with the goal to optimize the tumor removal (Figure 1c,d) [3]. However, the interpretation of the ultrasound can be complex [4,5]. The acquisition of the images through the skull opening, called craniotomy, requires skill and experience. Firstly, the sweep of the ultrasound probe is limited by the small opening. The presence of air between the probe and the brain surface blocks the propagation of ultrasound burst. Secondly, the scanned brain area in the images is limited. The orientation of the image and the interpretation of the information it shows can be complex, mostly for images acquired at the end of the operation.

A common method to assist the neurosurgeon in the visual analysis of the intraoperative ultrasound images is to visualize them overlapped to the preoperative MR data in the navigation system because the preoperative image data offers an overview of the entire head [2,6,7]. This technique has limitations for ultrasound images acquired at the end of the operation [5]. The brain structures are so largely deformed, mainly because of tissue resection, so that the information included in the visualization planes does not correspond at all anymore [8].

The evaluation and correction of the brain shift has been extensively studied. Methods are based on similarity measures based on image intensities [9,10,11,12], anatomical landmarks [13,14,15] or biomedical deformation models [16,17]. An interesting previous work proposed to use the vascular structures surrounding the tumor as landmarks in order to estimate the brain deformations [14,18]. In this work, the extraction of the blood vessels was performed in preoperative MR angiographic (MRA) data and in intraoperative Doppler ultrasound images. MRA examination is in routine not acquired in case of brain tumor. Doppler ultrasound can be performed intraoperatively but has limitations to depict the diameters with accuracy and to provide 3D visualization. Moreover, information about brain tissue itself is missing. Several medical studies were conducted to evaluate contrast-enhanced ultrasound imaging for the visualization of brain tumors and the detection of residuals of tumor [19,20,21]. This imaging modality enables to enhance vascularized structures, like lesions and tumors, but also blood vessels [22,23].

In this paper, an alternative method of vascular structures identification in contrast-enhanced ultrasound data is presented with a possible application in the brain shift estimation. Instead of considering the preoperative MRA and intraoperative Doppler ultrasound as previously mentioned, this work introduces the possibility of using cT1MR and 3D-iCEUS data which are routinely involved in brain tumor operations. The method has three main steps *i.e.*, the selection of a vascular segment in cT1MR, the identification of this pattern firstly in 3D-iCEUS${}_{start}$, then in the 3D-iCEUS${}_{end}$. Vesselness-based segmentation methods and rigid registrations techniques were employed, ensuring a computing time compatible with an intraoperative use. Another contribution of this work is the evaluation of the method on CEUS images acquired at the end of the operation, when tissue deformations are large.

## 2. Materials and Methods

### 2.1. Patient Image Dataset

Tumor operations were guided by using a neuronavigation system (SonoNavigator, Localite, Sankt Augustin, Germany), including an AplioXG ultrasound device (Toshiba Medical Systems Europe, Zoetermeer, Netherlands) with 2D ultrasound transducers. At the beginning of the intervention, preoperative 3D contrast T1 Magnetic Resonance (cT1MR) data were registered with the patient’s head, based on anatomical landmarks. The procedure was then improved using a surface-based registration technique. During the operation and in addition to the acquisition of intraoperative B-mode ultrasound volumes (noted 3D-iUS), two 3D intraoperative contrast enhanced ultrasound (3D-iCEUS) data were acquired after the injection of 4.8 milliliter of an ultrasound contrast agent (SonoVue, Bracco s.p.a., Milan, Italy) at a rate of 3.0 mL/min using a syringe pump (ACIST VueJect, Bracco s.p.a, Milano, Italy) and using the contrast harmonic imaging (CHI) method. The size and density of the micro-bubbles in the SonoVue contrast agent was 1.9 ± 0.1 micromillimeter and 3.4 ± 0.5 micro-bubbles/mL as recently reported in [24]. The first volume, denoted 3D-iCEUS${}_{start}$, was obtained transdurally immediately after the craniotomy when the tumor was still entire, and the second one, denoted 3D-iCEUS${}_{end}$, was acquired after removing the tumor at the end of the surgery. The surgeon scanned the cerebral region of interest (ROI) with the ultrasound probe (large linear array transducer with a field of view of 38 mm, a range of frequencies of 4.8 MHz to 11.0 MHz and an average frequency of 8 MHz was selected for the acquisitions). The transducer was tracked. The neuronavigation system reconstructs a 3D volume from the 2D-iCEUS slices and overlaid it onto the cT1MR data. The pixel size in the original 2D ultrasound images is 0.422 mm × 0.422 mm and the voxel size of the reconstructed 3D-iCEUS data is 1×1×1 mm${}^{3}$. The data involved in the surgery process are shown in Figure 1.

### 2.2. Vascular Structure Segmentation

A common solution to distinguish the blood vessels in the 3D-iCEUS data is their extraction by using segmentation methods. However, the segmentation of vascular structures in ultrasound image data is generally a complex task because of the speckle and the blood vessel diameters less than 2 millimeters (Figure 1b,d). Model-based techniques, which include a priori knowledge of the object shape or its data representation, are required to guide the segmentation process and improve the success of algorithms [25,26]. In this work, the extraction of blood vessels is performed in two steps: the computation of a Hessian based vesselness measure for enhancing the vascular structures, followed by the Otsu thresholding algorithm [27]. Moreover, the segmentation is performed in limited target regions of interest in the 3D-iCEUS data, as it will be explained in Section 2.3. The vesselness measure, which is used, was introduced by Sato [28,29] to describe tubular structures within an image using the Hessian matrix. The Hessian matrix *H* of an image *I* is defined by:

(1) $$H=\left[\begin{array}{ccc} \frac{\partial^{2}I}{\partial x^{2}} & \frac{\partial^{2}I}{\partial x\partial y} & \frac{\partial^{2}I}{\partial x\partial z}\\ \frac{\partial^{2}I}{\partial x\partial y} & \frac{\partial^{2}I}{\partial y^{2}} & \frac{\partial^{2}I}{\partial y\partial z}\\ \frac{\partial^{2}I}{\partial x\partial z} & \frac{\partial^{2}I}{\partial y\partial z} & \frac{\partial^{2}I}{\partial z^{2}} \end{array}\right]$$

The second order derivative of the image *I* is computed from convolution with a Gaussian kernel of standard deviation *σ*.

(2) $$I_{\sigma }=I*G_{\sigma }$$

where $G_{\sigma }$ is a Gaussian function with a standard deviation *σ*.

(3) $$G_{\sigma }=\frac{1}{\sqrt{{\left(2\pi \sigma^{2}\right)}^{3}}}\exp\left(-\frac{x^{2}+y^{2}+z^{2}}{2\sigma^{2}}\right)$$

Then, the multiscale Hessian matrix will be computed as:

(4) $$H_{\sigma }=\nabla^{2}I_{\sigma }$$

Based on experimental analysis of an ideal tube model, Sato *et al.* [28,30,31] proposed a vessel enhancement filter function given by

(5) $$v\left(\sigma \right)=\left\{\begin{array}{ccc} 0 & if\;\lambda_{c}=0 & \\ \exp\left(\frac{-\lambda_{1}^{2}}{2{\left(\alpha_{1}\lambda_{c}\right)}^{2}}\right).\lambda_{c} & if\;\lambda_{1}\leq 0,\lambda_{c}\neq 0 & \\ \exp\left(\frac{-\lambda_{1}^{2}}{2{\left(\alpha_{2}\lambda_{c}\right)}^{2}}\right).\lambda_{c} & if\;\lambda_{1}>0,\lambda_{c}\neq 0 & \end{array}\right.$$

where $\alpha_{1}<\alpha_{2}$ and $\lambda_{c}$ = $\min(-\lambda_{2},-\lambda_{3})$. The values of $\alpha_{1}$ and $\alpha_{2}$ are 0.5 and 2, respectively; and $\lambda_{1}\geq \lambda_{2}\geq \lambda_{3}$ (eingenvalues of the Hessian matrix).

Moreover, it has been shown that the variation of sigma values *σ* enables to describe vascular structures with different radii sizes. Therefore, v(σ) is computed at each voxel position in the image for different values of the standard deviations $\sigma_{min}\leq \sigma \leq \sigma_{max}$ and the maximum response kept $v_{I}$ is described by:

(6) $$v_{I}=\max_{\sigma_{min}\leq \sigma \leq \sigma_{max}}v(\sigma )$$

**Figure 2:**
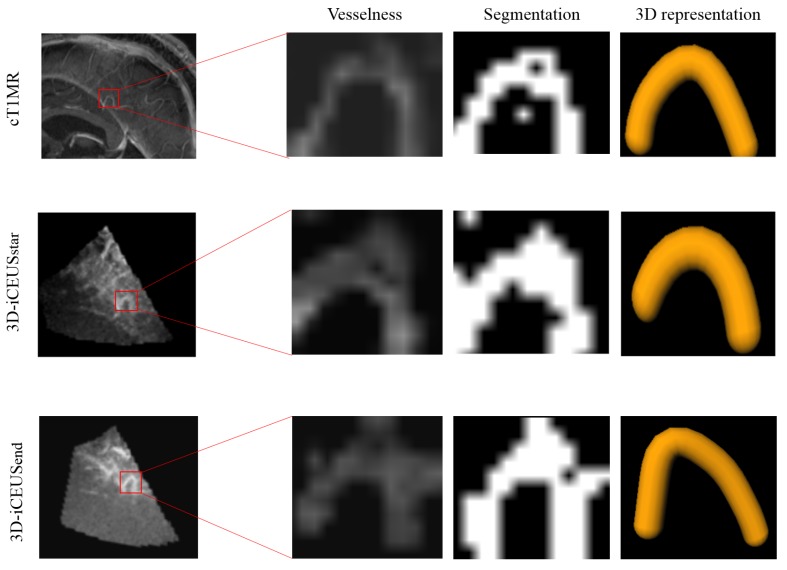
Illustration of vascular structure segmentation in cT1MR, 3D-iCEUS${}_{start}$ and 3D-iCEUS${}_{end}$.

Finally the obtained image of vesselness responses is thresholded using the Otsu method [27] in order to extract the vascular structures. Figure 2 presents an illustration of blood vessel segmentation process from cT1MR, 3D-iCEUS${}_{start}$ and 3D-iCEUS${}_{end}$. An additional step of 3D representation is added here.

### 2.3. Vascular Structure Identification

**Figure 3:**
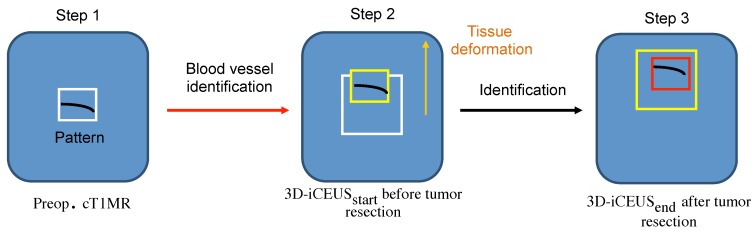
Process of the proposed vascular structure identification.

**Figure 4:**
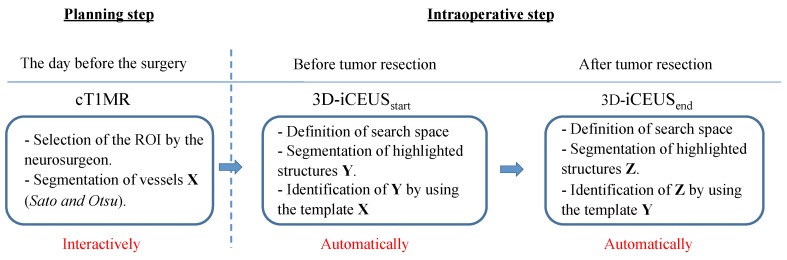
Workflow of the proposed method.

The proposed method consists in identifying the vascular structures in the image data involved in the patient treatment. During the planning stage, some vascular structures close to the tumor are selected by the neurosurgeon. These patterns are automatically recognized in the 3D-iCEUS data by using image registration techniques. The identification method of vascular structures is depicted in Figure 3 and is described as follows:

- Step 1: Selecting a vascular segment pattern in cT1MR
  Interaction with the application in the operating room has to be limited because of sterilization constraints and restricted time. Due to this, during the operation planning, the user delineates interactively a region of interest including a blood vessel near to the tumor in the cT1MR data. The vascular structure is segmented using the method described in Section 2.2. It performs well because the blood vessels are enhanced in the cT1MR data due to the contrast agent, and any other anatomical structure represented with similar intensities is included in the region of interest. The segmented blood vessel represents the pattern (white frame, Step 1, Figure 3) that is searched for in the 3D-iCEUS${}_{start}$ and 3D-iCEUS${}_{end}$ data.
- Step 2: Blood vessel identification in 3D-iCEUS${}_{start}$
  The pattern is firstly searched in the 3D-iCEUS${}_{start}$ data acquired before resection. In order to reduce the computing time, the search space (large white frame, Step 2, Figure 3) is smaller than the entire 3D-iCEUS${}_{start}$, but large enough to take the tissue deformations into account. It has twice the volume of the region of interest defined in the cT1MR data, and is centered on the same image position. The enhanced structures in this region are segmented and then a rigid registration method is used to find the sample in the 3D-iCEUS${}_{start}$ data (yellow frame, Step 2, Figure 3) which corresponds best to the pattern. A rigid transformation is sufficient here, since the goal is the identification of the position of the blood vessel in the 3D-iCEUS${}_{start}$ data, which looks like the vascular segment pattern selected in the cT1MR data. The blood vessel detected in the 3D-iCEUS${}_{start}$ then becomes the new pattern, which has to be identified in the 3D-iCEUS${}_{end}$ data after resection.
- Step 3: Blood vessel identification in the 3D-iCEUS${}_{end}$
  A similar method as described in the step 2 enables to find the position of the target blood vessel (red frame, Step 3, Figure 4) within the search space (yellow frame, Step 3, Figure 3) defined in the 3D-iCEUS${}_{end}$ data acquired after tumor resection.

Finally, the preselected vessel in the cT1MR is overlapped on the 3D-iCEUS${}_{start}$ and 3D-iCEUS${}_{end}$ images for visualization purpose. Figure 4 illustrates the workflow of the entire method and illustrations of the blood vessel identification on patient data is shown in Section 3.

It should be noted that the method is so far not able to recognize an incorrect identification of the selected blood vessel. In this case, the user has to reposition interactively the area including the vascular segment corresponding to the pattern. However, the approach is to minimize the interaction between surgeon and computer in operating room because of the sterilization requirement.

### 2.4. Validation

One key point in image registration is the similarity measure. The Normalized Cross Correlation (NCC) and the Normalized Mutual Information (NMI) are typical similarity measures commonly used [32,33]. The NCC computes pixel-wise normalized cross-correlation between two images and usually aims at registering images of the same modality. The NMI describes how well one image can predict the other one and is, therefore, suitable for multi-modality registration. Robust and easier to interpret, the Normalized Gradient Field (NGF) measures a normalized distance between the gradients of the images to be registered [34,35]. Some works proposed calculating the similarity measures from binary data, arguing that the registration process is, in this way, accelerated [36,37,38,39]. These three similarity measures were used for comparison in the registration algorithm. In order to validate the method, a neurosurgeon visually checked that the vascular segments, automatically identified in the 3D-iCEUS${}_{start}$ and 3D-iCEUS${}_{end}$, corresponded to the blood vessel selected in the cT1MR data. Moreover, the results of the algorithm were quantitatively compared with a registration, interactively performed, using the Dice Similarity Index (DSI) and the Hausdorff distance. The DSI represents the percentage of overlapping of two objects. Its value is 1 if they overlap perfectly and 0 if they do not intersect at all. The Hausdorff distance represents the maximal distance between two objects. The computing time of the registration process was also measured for each similarity measure. The NCC, NMI and NCC measures were alternately computed by using the following equations:

(7) $$NCC=\frac{1}{\sigma_{x}\sigma_{y}}\sum_{i}\left(x_{i}-\bar{x}\right)\left(y_{i}-\bar{y}\right)$$

(8) $$NMI=\frac{H(X)+H(Y)}{H(X,Y)}$$

where $x_{i}$ and $y_{i}$ are respectively the set of points of images *X* (fixed image) and *Y* (moving image). In addition, $\bar{x}$ and $\bar{y}$ are the mean of *X* and *Y*; and $\sigma_{x}$ and $\sigma_{y}$ are their standard deviation. H(X) and H(Y) are the entropies of random variables *X* and *Y*, and H(X,Y) represents their joint entropy.

(9) $$n\left(I,x\right):=\left\{\begin{array}{cc} \frac{\nabla I(x)}{\left||\nabla I(x)|\right|} & \;\nabla I(x)\neq 0\\ 0 & \;otherwise \end{array}\right.$$

(10) $$d^{c}\left(Y,X\right)=||n\left(X,x\right)\times {n\left(Y,x\right)||}^{2}$$

(11) $$d^{d}\left(Y,X\right)=<n\left(X,x\right),n\left(Y,x\right){>}^{2}$$

where n(I,x) is a regularized normalized gradient field of a given image I(x). Then, $d^{c}\left(Y,X\right)$ and $d^{d}\left(Y,X\right)$ represent the distance between the gradients of images *X* and *Y*.

Besides, the Dice Similarity Index (DSI) and the Hausdorff distance are described as follows:

(12) $$D\left(X,Y\right)=\frac{2|X\cap Y|}{\left|X|+|Y\right|}$$

(13) $$d_{H}\left(X,Y\right)=\max\left\{\;\max_{x\in X}\min_{y\in Y}∥x-y∥,\;\max_{y\in Y}\min_{x\in X}∥y-x∥\;\right\}$$

where *X* is the fixed image and *Y* the moving image.

## 3. Results

The collection of data has been performed at the University Hospital, Department of Neurosurgery, University of Leipzig, Germany, in the context of a previous research project funded by the German Research Society (Deutsche Forschungsgemein-schaft) and accepted by the ethic commission of the University of Leipzig. The implementation was done with an Intel Celeron, 1.5 GHz and 2 GB of memory using MeVisLab tool. The manual registration was validated by the neurosurgeon of this institution. The identification algorithm was tested on ten datasets of patients who overcame a brain tumor operation. A simple vascular segment near to the tumor was selected in the preoperative cT1MR data.

Table 1 includes the DSI, Hausdorff distances and the processing time values computed between the segmented blood vessels in the cT1MR and 3D-iCEUS${}_{start}$ data and between the segmented blood vessels in the 3D-iCEUS${}_{start}$ and 3D-iCEUS${}_{end}$ data, both normalized with the scores obtained by the expert registration. The values followed by a star indicate that the algorithm failed to find the correct vascular segment. If it occurred before resection, the identification in the 3D-iCEUS${}_{end}$ data was not performed. It is clearly observed that the proposed approach was able to find correctly the targeted vascular structure with at least one similarity measure used.

Besides, Table 2 includes the mean DSI values and the mean Hausdorff distances, averaged on the successful cases, while Table 3 presents the mean computing time. The outcomes show that the NGF achieves the highest mean rate of registration than the NMI and NCC. Nevertheless, the last ones performs the registration in a shortest time.

**Figure 5:**
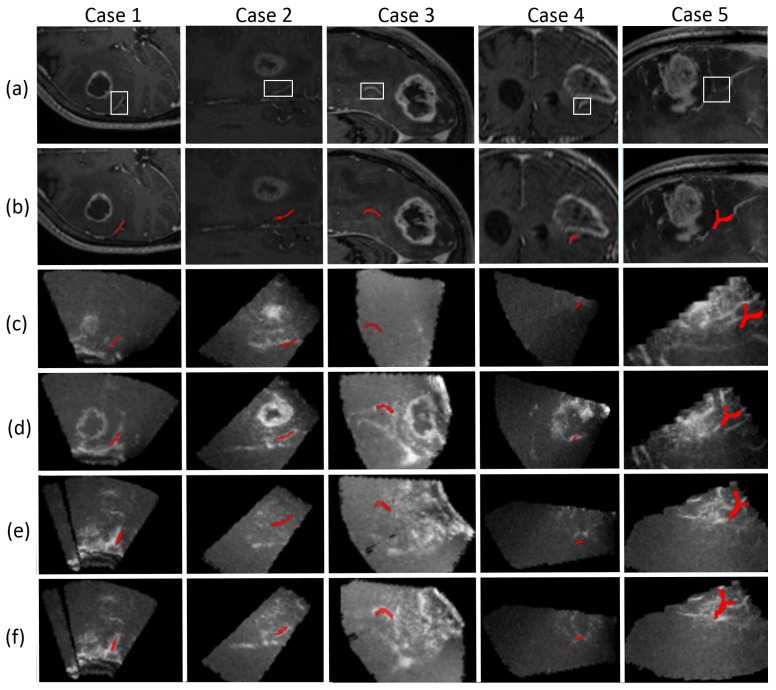
Illustration of vascular structures identification for five cases: (**a**) preoperative cT1MR; (**b**) vascular structure selection in cT1MR; (**c**) overlapping of vascular structure segmented in cT1MR on CEUS${}_{start}$ before registration; (**d**) overlapping of vascular structure segmented in cT1MR on CEUS${}_{start}$ after registration; (**e**) overlapping of vascular structure segmented in CEUS${}_{start}$ on CEUS${}_{end}$ before registration; (**f**) overlapping of vascular structure segmented in CEUS${}_{start}$ on CEUS${}_{end}$ after registration.

In Figure 5, the results obtained step by step for five cases are illustrated. First, the ROI is defined in the cT1MR by the surgeon (column (a)) in the planning stage and it encloses the selected vascular structure so as to reduce the space of segmentation. Second, the vessel is extracted by using the process described in Section 2.2 (column (b)). Third, the vascular structure extracted from the cT1MR data is overlaid on the 3D-iCEUS${}_{start}$ (column (c)). It should be observed that the extracted structure is not spatially aligned with its corresponding in the 3D-iCEUS${}_{start}$. Fourth, after the segmentation of the vascular structure in 3D-iCEUS${}_{start}$, column (d) presents the registration results, in which, vascular structures are aligned. At this step, the blood vessel found in 3D-iCEUS${}_{start}$ becomes the new pattern and it is superimposed on the 3D-iCEUS${}_{end}$ image in column (e). In most of cases, there is no matching between the new pattern with its corresponding vessel in the 3D-iCEUS${}_{end}$ because of large tissue deformations at this stage of the operation. Finally, the registration of vascular structures from the 3D-iCEUS${}_{star}$ to 3D-iCEUS${}_{end}$ is carried out (column (f)).

**Figure 6:**
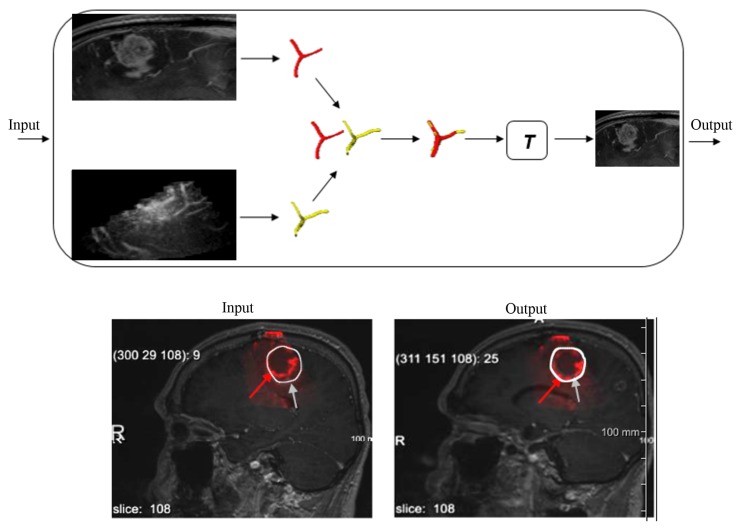
Application of vascular structure identification in brain shift correction between cT1MR and 3D-iCEUS${}_{start}$.

An illustration of using vascular structures from cT1MR and 3D-iCEUS${}_{start}$ for correcting the brain shift is depicted in Figure 6. First, the blood vessels are extracted from the both modalities and by overlapping them it is clearly observed that they are not aligned. Second, a registration process is performed to align the segmented structures. By registering the targets, a matrix denoted *T* which describes the spatial transformation necessary for aligning structures is obtained. Then, this transformation matrix is applied on the cT1MR images so as to achieve its matching with the 3D-iCEUS${}_{start}$ data. Finally, the superimposing of these image modalities is presented for the input before registration and for the output after registration.

## 4. Discussion

### 4.1. Visual Validation

The visual checking for identification a selected blood vessel was performed without problem in the 3D-iCEUS${}_{start}$. The ultrasound images were easy to interpret because the anatomical structures are, in general, similarly represented in the 3D-iCEUS${}_{start}$ and in the preoperative cT1MR data. Brain tissues were already moved at this stage of the operation (after the craniotomy) by comparing the images. The corresponding planes in the 3D-iCEUS${}_{start}$ and preoperative cT1MR data were coarsely only translated and the visual comparison of anatomical structures in these planes were still possible. After tumor resection, the comparison of the 3D-iCEUS${}_{start}$ and the 3D-iCEUS${}_{end}$ was much more complex for the expert. Firstly, the loss of tumor in the 3D-iCEUS${}_{end}$ deprives of a valuable reference structure for the comparison of the images. Secondly, the image quality was by trend lower in the 3D-iCEUS${}_{end}$. The acquisition was performed with the resection cavity filled with liquid in order to conduct the ultrasound waves. The liquid outflow outside the cavity caused artifacts in the images. Thirdly, brain tissues deformed largely between the beginning of the operation and the stage after tumor resection. Corresponding anatomical structures were elastically deformed. They were represented in different slices and the mental reconstruction of the information was difficult. Therefore, the postoperative MR image were important for assisting the visual evaluation.

### 4.2. Quantitative Validation

Additionally, the Dice similarity index and the Hausdorff distance were used for quantitatively assess the results obtained from the experiments. It was observed that the NGF similarity provided the highest mean DSI values and the lowest mean Hausdorff distances (Table 2). In contrast, it was found as the slowest (Table 2). Compared to the NMI and NCC, the particularity of the NGF measure is its robustness in cases of curved vascular segments. A T-test was performed to compare the DSI values obtained with the different similarity measures and it showed that the NGF and NCC are statistically different (*p*-value: 0.0304). As well as, the *p*-values obtained by comparing the NGF and NMI, then the NMI and NCC were 0.0611 and 0.1747, respectively. In general, the computing time is lower than 15 s on average. The calculation of the NCC and NMI involves the image intensities and was more quickly performed (computing time lower than 10 s on average, Table 3) while the NGF requires the previous computation of the image gradients (computing time lower than 13 s on average, Table 3). However, these mean computing time values are still acceptable for use in the operating theater. The size of the ROI plays an important role on the value of the computing time. It should be noticed that the results from the Table 1 show that the method will always perform a registration even if the correct vascular structure was not found. Despite the tissues deformation which tends to decrease the DSI value, a higher value than 0.60 was found acceptable compared to the neurosurgeon validation. However, the DSI close to 1 indicate the perfect matching obtained.

### 4.3. Limitations and Future Improvements

#### 4.3.1. Image Quality

The quality of the ultrasound images limits the performance of the registration algorithm. Mainly, it is affected by factors such as: the size of the craniotomy, the location and depth of the tumor in the brain that may complicate the acquisition process. Also, a precise sweeping of the 2D US probe has to be performed within a restricted time window, when the contrast agent enhances optimally the structures. The time window for an optimal acquisition is about 30 s [21]. Nevertheless, a technical solution to improve the image quality is the reduction of the size of voxel in the 3D ultrasound volume, for example 0.5 mm × 0.5 mm × 0.5 mm. The use a 3D ultrasound transducer will enable to perform the acquisition faster to overcome the washed in and out of contrast agent [40].

#### 4.3.2. Misidentification of Vascular Structures

The algorithm succeeded better if a vessel with a specific shape (e.g., curve) is selected. A single straight vascular segment can be in certain cases, confused with other vascular segments or with the resection cavity edges in the 3D-iCEUS${}_{end}$ data if they are close. Possible approaches to solve this problem would be to segment no-vascular structures in the images in order remove them in the registration process. For example, the tumor can be extracted using a model-based segmentation technique [41]. The resection cavity can be easier identified in the B-mode ultrasound images. Another interesting possibility to test in the future could be the use of the Scale-Invariant Feature Transform (SIFT) proposed by David Lowe [42,43] for registering images as applied in [44,45]. However, in spite of the difficulties mentioned above, the proposed methodology was able to find the correct vascular structures via at least one similarity measure used and, furthermore it was capable to carry out successfully the registration.

#### 4.3.3. Vascular Structures Segmentation

The traditional vesselness based on Hessian matrix was used in this study for vascular structure segmentation. The future intent could be, for instance, the use of alternative methods based on Gabor filter and multiobjective optimization applied in [46] on automatic segmentation of coronary arteries.

## 5. Conclusions

Despite the small size of the vascular structures surrounding the brain tumors, the low signal to noise ratio in ultrasound image and the brain tissue deformation, it was possible to correctly identify vascular segments in 3D-iCEUS patient’s data. Moreover, we showed that the NGF is more robust, especially in cases of vascular segments with specific shapes. However, the NCC and NMI have a lower computation time than the former. But it is important to notice that the computation time achieved by the algorithm by using these three similarity measures is still compatible with intraoperative use. Besides, an application of this work is the use of the vascular structures as landmarks for the estimation and correction of brain shift. The future works should be the improving of the vascular segmentation in 3D-iCEUS due to the low signal noise ratio in this modality in general. On the other hand, a solution has to be found in cases where the blood vessel is not visible in the intraoperative ultrasound data. For instance, the combination of information included in the B-mode and contrast-enhanced ultrasound data should make the method more robust.

## Figures and Tables

**Table 1:** Quantitative evaluation of the performance of three different similarity measures in the blood vessel registration algorithm using the DSI and the Hausdorff distance. The values in bold are the best obtained among the three similarity measures. * Indicate that the algorithm failed to find the correct vascular segment.

Patient	Similarity Measure	ROI in cT1MR (voxels)	Blood Vessel Identification in the 3D-iCEUS${}_{start}$ Using the Pattern from the cT1MR			Blood Vessel Identification in the 3D-iCEUS${}_{end}$ Using the Pattern from the 3D-iCEUS${}_{start}$		
			Processing Time (s)	DSI	Hausdorff Distance (mm)	Processing Time (s)	DSI	Hausdorff Distance (mm)
	NGF		2.8	0.89	8.083	3.5	0.824	12.207
1	NMI	50 × 29 × 8	2	0.903	7.874	2.8	0.937	11.180
	NCC		**1.8**	**0.982**	**7.483**	**1.7**	**0.994**	**10.770**
	NGF		11.1	0.645	21.772	14	0.813	11.180
2	NMI	19 × 37 × 26	**3.5**	**0.946**	**17.917**	2.3	**0.936**	**10.677**
	NCC		5.9	0.676	18.815	**2.1**	0.0 *	14.071 *
	NGF		3.1	0.976	5.385	1.3	**0.986**	**12.042**
3	NMI	35 × 16 × 13	2.9	**0.997**	**5.385**	**0.3**	0.263 *	21.237 *
	NCC		**0.9**	0.979	5.385	0.5	0.0 *	22.159 *
	NGF		5.7	0.766	12.369	1.5	0.829	9.487
4	NMI	15 × 17 × 22	**0.7**	**0.937**	**8.124**	**0.7**	**0.925**	**8.602**
	NCC		0.9	0.839	9.274	7.4	0.825	9.487
	NGF		2.1	**0.748**	**13.403**	13	**0.763**	**29.172**
5	NMI	61 × 55 × 8	2.2	0.422 *	28.792 *	**4.9**	0.38	34.015 *
	NCC		**1.2**	0.408 *	29.682 *	6.7	0.674	32.016
	NGF		**0.4**	0.0 *	22.023 *	-	-	-
6	NMI	8 × 39 × 26	1.8	**0.832**	**9.110**	**4.9**	**0.98**	**10.863**
	NCC		2.4	**0.832**	**9.110**	16.2	0.913	10.863
	NGF		17.6	**0.953**	**28.547**	56.6	**1**	**17.234**
7	NMI	49 × 56 × 29	11.3	0.827	29.653	30.2	0.051 *	19.519 *
	NCC		**9.1**	0.983	28.530	**8.8**	0.0 *	19.519 *
	NGF		3.2	**0.988**	**4.690**	**8.3**	**0.849**	**10.050**
8	MI	32 × 14 × 19	2.9	0.031 *	17.720 *	-	-	-
	NCC		**1.3**	0.097 *	15.524 *	-	-	-
	NGF		3.1	**0.988**	**14.000**	**4.1**	**0.879**	**14.000**
9	NMI	31 × 22 × 22	**2.5**	0.475 *	24.083 *	-	-	-
	NCC		3.5	0.458 *	25.729 *	-	-	-
	NGF		2.7	**0.952**	**2.449**	1.5	**0.961**	**3.162**
10	NMI	12 × 18 × 12	**0.7**	0.818	2.500	**0.7**	0.0 *	8.464 *
	NCC		2.4	**0.952**	2.449	1.8	0.0 *	8.718 *

**Table 2:** Mean DSI values and Hausdorff distances computed forthe three similarity measures and by comparison with the expert registration.

Comparative Studies	Mean DSI (Algorithm, Expert) Computed after Registration in the:		Mean Hausdorff Distances in mm (Algorithm, Expert) Computed after Registration in the:	
	3D-iCEUS${}_{start}$	3D-iCEUS${}_{end}$	33D-iCEUS${}_{start}$	3D-iCEUS${}_{end}$
NGF *vs.* Expert	**0.870**	**0.868**	**13.272**	**13.170**
NMI *vs.* Expert	0.838	0.832	15.116	15.570
NCC *vs.* Expert	0.815	0.852	15.198	15.950

**Table 3:** Mean processing time in *s* during the registration computed for the successful cases (patient 10 excluded).

Similarity Measure	cT1MR − 3D-iCEUS${}_{start}$	3D-iCEUS${}_{start}$ − 3D-iCEUS${}_{end}$
NGF	6.1	12.8
NMI	3.5	**3.1**
NCC	**3.2**	8.0

## References

[1] Unsgaard G., Rygh O., Selbekk T., Muller T., Kolstad F., Lindseth F., Nagelhus Hernes T. (2006). Intra-operative 3D ultrasound in neurosurgery. Acta Neurochir..

[2] Unsgaard G., Selbekk T., Muller T., Ommedal S., Torp S., Myhr G., Bang J., Nagelhus Hernes T. (2005). Ability of navigated 3D ultrasound to delineate gliomas and metastases—Comparison of image interpretations with histopathology. Acta Neurochir..

[3] Selbekk T., Jakola A., Solheim O.E.A. (2013). Ultrasound imaging in neurosurgery: Approaches to minimize surgically induced image artefacts for improved resection control. Acta Neurochir..

[4] Solheim O., Selbekk T., Jakola A., Unsgard G. (2010). Ultrasound-guided operations in unselected high-grade gliomas-overall results, impact of image quality and patient selection. Acta Neurochir..

[5] Selbekk T., Jakola A., Solheim O., Johansen T., Lindseth F., Reinertsen I., Unsgard G. (2013). Ultrasound imaging in neurosurgery: approaches to minimize surgically induced image artefacts for improved resection control. Acta Neurochir..

[6] Trantakis C., Meixensberger J., Lindner D., Straub G., Grunst G., Schmidtgen A., Arnold S. (2002). Iterative neuronavigation using 3D ultrasound. A feasibilty study. Neurol. Res..

[7] Lindner D., Trantakis C., Renner C., Arnold S., Schmitgen A., Schneider J., Meixensberger J. (2006). Application of Intraoperative 3D Ultrasound During Navigated Tumor Resection. Minim. Invasive Neurosurg..

[8] Maurer C.R., Hill D.L.G., Maciunas R.J., Barwise J.A., Fitzpatrick J.M., Wang M.Y. Medical Image Computing and Computer-Assisted Interventation. Proceedings of the MICCAI’98: First International Conference.

[9] Letteboer M., Willems P., Viergever M., Niessen W. (2005). Brain shift estimation in image-guided neurosurgery using 3-D ultrasound. IEEE Trans. Biomed. Eng..

[10] Ji S., Wu Z., Hartov A., Roberts D.W., Paulsen K.D. (2008). Mutual-information-based image to patient re-registration using intraoperative ultrasound in image-guided neurosurgery. Med. Phys..

[11] Coupe P., Hellier P., Morandi X., Barillot C. (2012). 3D Rigid Registration of Intraoperative Ultrasound and Preoperative MR Brain Images Based on Hyperechogenic Structures. Int. J. Biomed. Imaging.

[12] Fuerst B., Wein W., Muller M., Navab N. (2014). Automatic ultrasound-MRI registration for neurosurgery using the 2D and 3D {LC2} Metric. Med. Image Anal..

[13] Comeau R., Sadikot A., Fenster A., Peters T. (2000). Intraoperative ultrasound for guidance and tissue shift correction in image-guided neurosurgery. Med. Phys..

[14] Reinertsen I., Lindseth F., Unsgaard G., Collins D. (2007). Clinical validation of vessel-based registration for correction of brain-shift. Med. Image Anal..

[15] Hartov A., Roberts D., Paulsen K. (2008). A comparative analysis of coregistered ultrasound and magnetic resonance imaging in neurosurgery. Neurosurgery.

[16] Ferrant M., Nabavi A., Macq B., Jolesz F., Kikinis R., Warfield S. (2001). Registration of 3-D intraoperative MR images of the brain using a finite-element biomechanical model. IEEE Trans. Med. Imaging..

[17] Hawkes D., Barratt D., Blackall J., Chan C., Edwards P., Rhode K., Penney G., McClelland J., Hill D. (2005). Tissue deformation and shape models in image-guided interventions: A discussion paper. Med. Image Anal..

[18] Reinertsen I., Lindseth F., Askeland C., Iversen D.H., Unsgard G. (2014). Intra-operative correction of brain-shift. Acta Neurochir..

[19] Hansen C., Wilkening W., Ermert H., Engelhardt M., Schmieder K., Krogias C., Eyding J. Intraoperative contrast enhanced perfusion imaging of cerebral tumors. Proceedings of the 2005 IEEE Ultrasonics Symposium.

[20] Kanno H., Ozawa Y., Sakata K., Sato H., Tanabe Y., Shimizu N., Yamamoto I. (2005). Intraoperative power Doppler ultrasonography with a contrast-enhancing agent for intracranial tumors. J. Neurosurg..

[21] Prada F., Perin A., Martegani A., Aiani L., Solbiati L., Lamperti M., Casali C., Legnani F., Mattei L., Saladino A. (2014). Intraoperative contrast-enhanced ultrasound for brain tumor surgery. Neurosurgery.

[22] Holscher T., Ozgur B., Singel S., Wilkening W., Mattrey R., Sang H. (2007). Intraoperative ultrasound using phase inversion harmonic imaging: first experiences. Neurosurgery.

[23] Prada F., Bene M., Saini M., Ferroli P., DiMeco F. (2015). Intraoperative cerebral angiosonography with ultrasound contrast agents: How I do it. Acta Neurochir..

[24] Hyvelin J.M., Greis C., Gaud E., Costa M., Helbert A., Bussat P., Bettinger T., Frinking P. Characteristics and echogenicity of clinical ultrasound contrast agents: An *in vitro* and *in vivo* comparison study. Proceedings of the 21 European Symposium on Ultrasound Contrast Imaging, An ICUS Conference, Erasmus MC Rotterdam.

[25] Gill J., Ladak H., Steinman D., Fenster A. (2000). Accuracy and variability assessment of a semiautomatic technique for segmentation of the carotid arteries from three-dimensional ultrasound images. Med. Phys..

[26] Chalopin C., Krissian K., Meixensberger J., Muns A., Arlt F., Lindner D. (2013). Evaluation of a semi-automatic segmentation algorithm in 3D intraoperative ultrasound brain angiography. Biomed. Tech..

[27] Otsu N. (1979). A Threshold Selection Method from Gray-Level Histograms. IEEE Trans. Syst. Man Cybern..

[28] Yoshinobu S., Shin N., Atsumi H., Thomas K., Guido G., Shigeyuki Y., Ron K. CVRMed-MRCAS’97: First Joint Conference Computer Vision. Proceedings of the Virtual Reality and Robotics in Medicine and Medical Robotics and Computer-Assisted Surgery.

[29] Sato Y., Nakajima S., Shiraga N., Atsumi H., Yoshida S., Koller T., Gerig G., Kikinis R. (1998). Three-dimensional multi-scale line filter for segmentation and visualization of curvilinear structures in medical images. Med. Image Anal..

[30] Luu H.M., Klink C., Moelker A., Niessen W., van Walsum T. (2015). Quantitative evaluation of noise reduction and vesselness filters for liver vessel segmentation on abdominal CTA images. Phys. Med. Biol..

[31] Drechsler K., Laura C.O. Comparison of vesselness functions for multiscale analysis of the liver vasculature. Proceedings of the 2010 10th IEEE International Conference on Information Technology and Applications in Biomedicine (ITAB).

[32] Andronache A., von Siebenthal M., Székely G., Cattin P. (2008). Non-rigid registration of multi-modal images using both mutual information and cross-correlation. Med. Image Anal..

[33] Pluim J., Maintz J., Viergever M. (2003). Mutual-information-based registration of medical images: A survey. IEEE Trans. Med. Imaging.

[34] Hodneland E., Lundervold A., Rorvik J., Munthe-Kaas A.Z. (2014). Normalized gradient fields for nonlinear motion correction of DCE-MRI time series. Comput. Med. Imaging Graph..

[35] Haber E., Modersitzki J. (2005). Bildverarbeitung für die Medizin 2005: Algorithmen—Systeme—Anwendungen Proceedings des Workshops vom 13.–15. März 2005 in Heidelberg.

[36] Bogush R., Maltsev S., Ablameyko S., Uchida S., Kamata S. An efficient correlation computation method for binary images based on matrix factorisation. Proceedings of the Sixth International Conference on Document Analysis and Recognition.

[37] Chanwimaluang T., Fan G., Fransen S. (2006). Hybrid retinal image registration. IEEE Trans. Inf. Technol. Biomed..

[38] Sun M., Qiao G., Zhang R., Zong G. Characteristics of Independence on Image Gray Level in NCCO Applications. Proceedings of the International Conference on Information Technology and Computer Science (ITCS 2009).

[39] Crabb M.G., Davidson J.L., Little R., Wright P., Morgan A.R., Miller C.A., Naish J.H., Parker G.J.M., Kikinis R., McCann H. (2014). Mutual information as a measure of image quality for 3D dynamic lung imaging with EIT. Physiol. Meas..

[40] Müns A., Meixensberger J., Arnold S., Schmitgen A., Arlt F., Chalopin C., Lindner D. (2011). Integration of a 3D ultrasound probe into neuronavigation. Acta Neurochir..

[41] Chalopin C., Lindenberg R., Arlt F., Muns A., Meixensberger J., Lindner D. (2012). Brain tumor enhancement revealed by 3D intraoperative ultrasound imaging in a navigation system. Biomed. Eng./Biomed. Tech..

[42] Lowe D.G. Object recognition from local scale-invariant features. Proceedings of the Seventh IEEE International Conference on Computer Vision.

[43] Lowe D.G. (2004). Distinctive Image Features from Scale-Invariant Keypoints. Int. J. Comput. Vis..

[44] Ghassabi Z., Shanbehzadeh J., Sedaghat A., Fatemizadeh E. (2013). An efficient approach for robust multimodal retinal image registration based on UR-SIFT features and PIIFD descriptors. EURASIP J. Image Video Process..

[45] Chen J., Tian J. (2009). Real-time multi-modal rigid registration based on a novel symmetric-SIFT descriptor. Progress Natural Sci..

[46] Cruz-Aceves I., Oloumi F., Rangayyan R.M., Avina-Cervantes J.G., Hernandez-Aguirre A. (2016). Automatic segmentation of coronary arteries using Gabor filters and thresholding based on multiobjective optimization. Biomed. Signal Process. Control.

